# Mechanosensitive Ion Channels and Their Role in Cancer Cells

**DOI:** 10.3390/membranes13020167

**Published:** 2023-01-29

**Authors:** Julia Karska, Szymon Kowalski, Jolanta Saczko, Mihaela G. Moisescu, Julita Kulbacka

**Affiliations:** 1 Faculty of Medicine, Wroclaw Medical University, 50-345 Wroclaw, Poland; 2 Department of Molecular and Cellular Biology, Faculty of Pharmacy, Wroclaw Medical University, 50-556 Wroclaw, Poland; 3 Department of Biophysics and Cellular Biotechnology, Research Center of Excellence in Biophysics and Cellular Biotechnology, Faculty of Medicine, Carol Davila University of Medicine and Pharmacy, 050474 Bucharest, Romania; 4 Department of Immunology, State Research Institute Centre for Innovative Medicine, 08406 Vilnius, Lithuania

**Keywords:** mechanosensitive ion channels, piezo channels, mechanoreceptors, cancer

## Abstract

Mechanical forces are an inherent element in the world around us. The effects of their action can be observed both on the macro and molecular levels. They can also play a prominent role in the tissues and cells of animals due to the presence of mechanosensitive ion channels (MIChs) such as the Piezo and TRP families. They are essential in many physiological processes in the human body. However, their role in pathology has also been observed. Recent discoveries have highlighted the relationship between these channels and the development of malignant tumors. Multiple studies have shown that MIChs mediate the proliferation, migration, and invasion of various cancer cells via various mechanisms. This could show MIChs as new potential biomarkers in cancer detection and prognosis and interesting therapeutic targets in modern oncology. Our paper is a review of the latest literature on the role of the Piezo1 and TRP families in the molecular mechanisms of carcinogenesis in different types of cancer.

## 1. Introduction

Cancers are undoubtedly one of the most urgent problems in present and future medicine [1,2]. According to the WHO, by 2030, malignant tumors will cause 80% of deaths worldwide [3,4]. Nowadays, malignant cancers are characterized by the highest mortality rates among all diseases [5,6,7]. Cancer development depends strongly on complex molecular processes connected to the genome, such as changes in oncogenes activity [8], and is influenced by both exo- and endogenous factors [9] that lead to genetic instability of the cell. Although there are considerably more new and performant diagnostic and treatment methods for malignant tumors [10,11], they are not enough to cope with these pathologies efficiently. Therefore, it is important to extend the research activities dedicated to molecular and cell biology of cancer cells; in recent years, more and more scientists have been interested in mechanosensitive ion channels (MIChs). These channels are associated with various important biological processes in many types of human cells, including tumor cells. Mechanosensitization is necessary to feel skin contact, gravity, proprioception, sound waves, food texture, muscle stretch, and air flow. The activation of MIChs requires an application of a mechanical force subject to further transduction into a chemical or electrical signal [12].

## 2. Evidence Acquisition

For the purposes of this narrative review, we conducted comprehensive English language literature research for original and review articles using the PubMed and Scopus databases through September 2022. We searched for the combination of the following terms: Piezo channels (Piezo); Piezo in cancers; transient receptor potential channels (TRP); TRPM (melastin); TRPV (vanilloid); TRPC (canonical). We found 2608 related articles, and the final number of papers that were selected for this manuscript was 159. Studies with the highest level of evidence and relevance to the discussed topics (32) were selected by the authors’ consensus.

## 3. The Overview of Mechano-Sensitive Ion Channels

Mechano-gated ion channels are a unique class of channels that respond to mechanical forces such as pressure, stretch, or touch. These channels play a crucial role in various physiological processes, including sensation, muscle contraction, and blood pressure regulation. In the neurosensory field, mechano-gated ion channels are particularly important for the detection of touch and pressure, hearing, and the function of other mechanical senses [11].

There are several families of mechanosensitive ion channels that have been identified, including Piezo or TRP (transient receptor potential) among others. These channels have different properties and functions and are activated by different mechanical stimuli. For example, Piezo channels are activated by mechanical stretching of the cell membrane, while TRP channels are activated by changes in temperature, pressure, or chemical stimuli [12].

Mechanically activated ion channels, such as MscL, MscS, TREK-1, Piezo1, OSCA1.2, TRP, and NOMPC (neuronal mechanosensitive cation channel), share a similar structural feature—the presence of amphipathic helices, which are amino acid segments with both hydrophobic and hydrophilic regions. These amphipathic helices are connected directly or indirectly to the pore-lining regions of the channels. The structure of MscL, for example, has been determined using X-ray crystallography and is composed of a pore-lining transmembrane segment (TM1) connected to an amphipathic S1 helix [13,14]. The structure of MscS, on the other hand, has been determined using electron microscopy and X-ray crystallography, and the recent structure shows an amphipathic anchor domain sitting on the external membrane leaflet, while the previous model showed the pore-lining TM3a helix embedded within the membrane and TM3b as an amphipathic segment at the cytoplasmic leaflet [14,15].

The structure of TREK-1 is composed of pore domains that are gated by a C-type mechanism, with an amphipathic C-tail extending below the M4 helix [16]. Piezo1’s structure is composed of two transmembrane helices from each subunit that line the pore and several amphipathic helices that line the cytoplasmic leaflet [17,18,19,20]. OSCA1.2’s structure has five helices that line each of the two putative pores, and an amphipathic helix sits on the opposite face of each subunit [21,22,23,24]. TRP structure has canonical tetrameric architecture, with cytosolic N- and C-termini and six TM domains [25]. NOMPC’s structure is composed of an amphipathic TRP domain, a pore helix, and a large spring-like ankyrin repeat domain in each subunit [26].

Another type of mechanosensitive channel is Tmc, also known as transmembrane channel-like 1 (TMC1) and TMC2, as a key component of the auditory system, responsible for converting mechanical vibrations into electrical signals that can be interpreted by the brain.

Recent research has focused on understanding the systematic properties of Tmc, including its structure, function, and regulation. TMC-1 is composed of two subunits, TMC-1A and TMC-1B. The structure of TMC-1 was determined using cryo-electron microscopy (cryo-EM) and X-ray crystallography. The TMC-1A subunit is composed of two transmembrane helices, TM1 and TM2, and an extracellular domain that forms a pore through which ions can flow. The TMC-1B subunit is composed of a single transmembrane helix, TM3, and an intracellular domain that is involved in signaling [27]. Additionally, Tmc is regulated by a variety of signaling pathways, including the Rpn4 proteotoxic stress response [28].

While much of the research on Tmc has focused on its role in the auditory system, recent studies have also investigated its expression and potential role in cancer cells. Some studies have suggested that Tmc may be overexpressed in certain types of cancer, such as lung and breast cancer, and may play a role in cell proliferation and migration [29]. However, more research is needed to fully understand the relationship between Tmc and cancer and to determine whether targeting Tmc may be a viable therapeutic strategy for cancer treatment [30].

**Figure 1:**
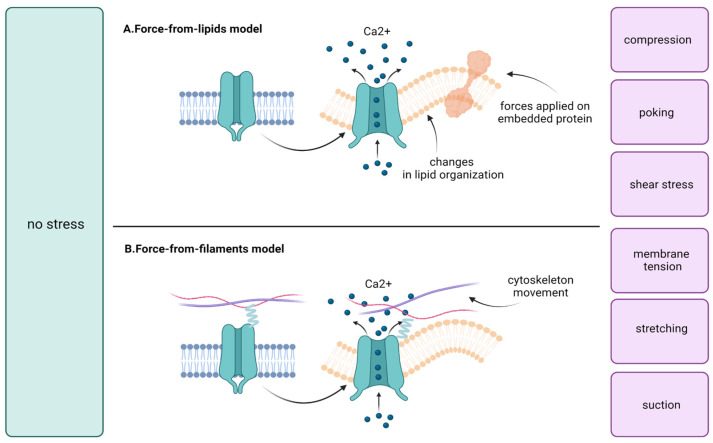
Mechanical stimuli, e.g., compression, membrane tension, poking, shear stress, stretching, or suction activates calcium influx through mechanosensitive ion channels. Then, calcium acts as a signal molecule on various pathways which may lead to, e.g., cell proliferation [43,44,45,46]. Two models of mechanosensitive ion channel gating have been established. (**A**) In the force-from-lipids model, alterations in lipid organization lead to channel activation; (**B**) In the force-from-filaments model, the causative factors are changes in the extracellular matrix and cytoskeleton [31,32,33,34,35,36,37,38].

According to activation, two mechanisms have been established for mechanosensitive ion channels: the force-from-lipids model and the force-from-filaments model (Figure 1). The first one assumes that membrane-bound mechanosensitive ion channels are subjected to changes in tension or stretching of the lipid bilayer. The response to these stimuli leads to the induction of conformational changes and the opening of the channel [31]. The force-from-lipids model was first proposed in conjunction with the activation of bacterial mechanosensitive ion channels by amphipaths in giant spheroplasts of Escherichia coli [32]. A similar phenomenon has also been described for eukaryotic channels such as two-pore domain potassium channels TREK-1 and TRAAK, TRPV4, and Piezo1 [31,32,33,34]. In the force-from-filaments model a membrane-bound channel is elastically tethered between the cytoskeleton and the extracellular molecules, and force-induced displacement of these tethers opens the channel [35]. This type of activation is hypothesized for some members of the TRP and ASIC family and Piezo2 [36,37,38].

Another important aspect of mechanosensitive ion channels is their high sensitivity to changes in matrix stiffness. The cellular microenvironment is crucial in tumorogenesis and metastasis. In tumor development, mechanical cues, acting via mechanosensitive ion channels, affect both the cancer cells directly and the microenvironment as well. These signals are transduced into a cellular response (e.g., proliferation) or to a response affecting the matrix microenvironment (e.g., inducing fibrosis or building up growth-induced pressure) [39,40]. A change in tissue mechanical properties is a physical hallmark of many solid tumors. It has been shown that alteration in the matrix microenvironment may lead to overexpression of some types of MIChS such as Piezo1. Chen et al. described this relationship for glioma and discovered that Piezo regulates mitosis and tissue rigidity of the tumor. Piezo1 occurs at focal adhesions and activates integrin–FAK signaling, regulates the extracellular matrix, and reinforces tissue stiffening. Moreover, the matrix alteration leads to Piezo1 overexpression, creating the bidirectional relationship [41]. The structure or gating of mechanosensitive ion channels and their relations with the matrix are not yet fully understood and further research is needed. Nevertheless, the current knowledge of these channels has important implications for oncology among others, especially according to the role of Piezo and TRP channels [42,43,44].

## 4. Piezo Channels

### 4.1. Introduction to Piezo 

Piezo are mechanically activated (MA) non-selective Ca^2+^-permeable channels that transduce diverse mechanical forces into biochemical signals [47]. These channels can be classified into two types: Piezo1 and Piezo2 which differ in both structure and function [48]. Both Piezo are trimeric propeller-shaped channel proteins with a central anchor, three long beams, and three blade-like structures. The central part is a cation-conducting channel formed by two transmembrane helices with the three curved blades surrounding the pore, resulting in a structure that locally deforms the cell membrane [18,49,50,51,52]. This deformation is triggered by mechanical stimuli such as compression, stretching, poking, shear stress, membrane tension, and suction; in particular, Piezo2 is triggered by light touch, pain, and proprioception [43,44,45,46]. It has been shown that the Piezo1 gene is homologous in humans, zebrafish, chickens, fruit flies, birds, mice, paramecia, and African clawed toads [40]. However, it does not bear sequence homology with any other known class of ion channels found in mammals [53]. Piezo1 is widely found in tissues of the skin, lungs, bladder, digestive tract, and the lining of blood vessels, but it is less expressed in the cerebellum and skeletal muscles. Piezo2 channels are highly expressed in dorsal root and trigeminal ganglia sensory neurons, the lungs, bladder, colon, and Merkel cells and less abundant in the cerebellum, kidneys, or heart [48,54,55]. 

Recently, the roles of Piezo channels in human physiology have been deepened by registering the consequences of mutations in Piezo genes. Abnormalities due to Piezo1 gene mutations are reported in generalized lymphatic dysplasia, dehydrated stomatocytosis, hereditary xerocytosis, heart failure, and diabetes mellitus [56,57,58,59,60,61], whereas the pathologies caused by Piezo2 gene mutations are Gordon syndrome, Marden–Walker syndrome, and scoliosis [62,63]. Aberrant expressions of both Piezo1 and Piezo2 genes are in many ways linked to tumor development and metastases.

### 4.2. Breast Cancer

In breast cancer cells, functional mechanosensitive ion channels (of Piezo1 type) were discovered for the first time in an MCF cell line by Li C et al. [64]. However, it was found that Piezo1 is also expressed in normal breast cells, but a higher expression characterizes cancer cells [65,66]. Cell membrane regions especially rich in Piezo1 are the caveolae, which present a high colocalization of mechanosensitive ion channels. Knocking down the caveolin1 expression or depleting the cholesterol content of the membrane reduces the number of caveolae and inhibits the expression of Piezo1. The Piezo1 dependent on caveolae is responsible for both the invasion and migration of breast cancer cells MDA-MB-231 [64]. Therefore, one may consider that a high level of mRNA for Piezo1 decreases the patient’s survival rate [1].

**Figure 2:**
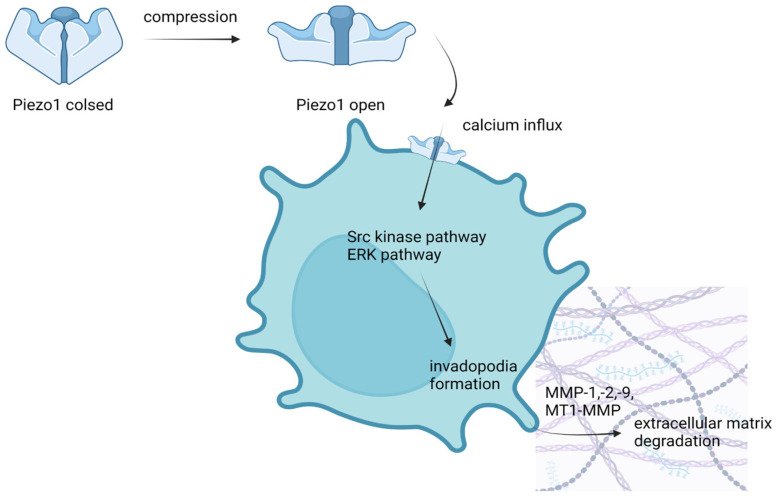
Compression exerted on a breast cancer cell activates Piezo channels through which calcium flows in. Then, the ion induces activation of both Src and ERK pathways that cause the formation of invadopodia in the cell membrane. Invadopodia destroys the extracellular matrix and, thus, promotes cell migration [64,66].

Piezo1 induces both collective and single-cell migration by modulating cells’ adhesion, stiffness, and contractility [66]. The dynamics of the F-actin cytoskeleton as well as focal adhesions regulate the relationship between a cell and its matrix by supporting cell stiffness and contractility. Piezo1 disturbs these processes leading to greater cancer cell motility. When it comes to metastasis capability, Piezo1 induces migration of confined cancer cells but inhibits that of unconfined ones. Piezo1’s contribution to cancer cell invasion is also ensured by calcium signal initiation, actin protrusion formation (invadopodia), and enhanced matrix degradation [64,66]. Mechanical activation by compression of Piezo channels induces calcium influx which stimulates downstream signaling pathways such as Src and extracellular regulated protein kinase (ERK), finally leading to the formation of actin-based protrusions called invadopodia. Invadopodia are able to degrade the surrounding extracellular matrix by metalloproteinases (MMP-1,-2,-9, MT1-MMP) which are cumulated in these protrusions (Figure 2). Moreover, Piezo1 causes inhibition of fibroblast formation and therefore supports even more invasion and migration of cancer cells [66]. The role of Piezo1 in modifying the proliferation rate and cell cycle of breast cancer cells is controversial. Yu Y et al. claim no significant influence of Piezo on proliferation and the cell cycle [66], whereas Luo M et al. showed that repetitive compression enhanced cancer cell invasion through activation of Piezo1 which promoted cell proliferation [64].

### 4.3. Oral Cancer

Piezo1 plays an important role in the proliferation of oral squamous cell carcinoma (OSCC). When Piezo1 expression is upregulated, the proliferative feature of the tumor is promoted [67]. This correlation is inherently attributed to the Hippo pathway and YAP signaling. The inactivation of the Hippo pathway results in nuclear translocation of the transcription co-activators: the yes-associated protein (YAP) and transcriptional co-activator with PDZ-binding motif (TAZ) [68,69,70,71,72,73,74,75]. Hasegawa K et al. revealed that Piezo1 is a transcriptional target of YAP in OSCC, and Piezo1 upregulated expression leads to Piezo1 agonist-dependent Ca^2+^ influx which consequently activates ERK1/2 and p38 MAPK that are involved in cell proliferation. Moreover, the presence of Ki-67 and PCNA (proliferation markers) in the cell is in direct proportion with Piezo1 expression. However, Piezo1 expression induction through YAP signaling is not relevant for the clinical T (tumor size) stage, histological grade, or lymph node metastasis [67].

### 4.4. Laryngeal Squamous Cell Carcinoma

Not Piezo1, but Piezo2 and the hypermethylation of its promoter support the development of laryngeal squamous cell carcinoma (LSCC). In tumor tissues, Piezo2 promoter hypermethylation is significantly more frequent than in normal tissues and correlates with gender, differentiation, tumor (T) stage, lymph node metastasis, and clinical stage. Consequently, Piezo2 promoter hypermethylation is not only an LSCC risk factor but also a marker for tumor progression and the presence of metastases, a prediction marker of a patient’s poorer survival, and a possible therapeutic target [76].

### 4.5. Gastric Cancer

Piezo1 expression is upregulated in the majority of gastric cancer cell lines, especially those metastasizing to the omentum and lymph nodes, causing poor disease-specific survival [77,78]. There are reports stating that Piezo1 high expression correlates with both lymphatic metastasis and TNM staging of gastric cancer [78]. In this cancer, Piezo1 enhances cell proliferation and motility and, therefore, cancer migration and invasion. The mechanisms regulating these processes are numerous [77,79]. It was discovered that Piezo1 is a novel TFF1 (trefoil factor family 1) binding protein [79]. TFF1 is responsible for epithelial restitution and cell motility. It is mainly expressed in epithelial gastric cells and its expression is increased during inflammation. Through cell migration to the wound and angiogenesis, TFF1 repairs the lesion. These features of Piezo1-TFF1 are also very much involved in tumorigenesis. By using a wound healing assay, Yang X.N. et al. showed that Piezo1 knockdown inhibits gastric cancer cell migration and reduces the pro-migratory effect of TFF1 [79]. It was discovered that TFF1 binds to the C-domain of Piezo1, however, it does not cause Ca^2+^ influx. This signaling pathway remains unknown. Piezo1 knockdown cells also presented a decreased expression of integrin β1. This type of integrin has been reported to be highly expressed in malignant phenotypes of gastric cancer and supports cell migration [80,81,82]. Another mechanism of Piezo1’s positive influence on cell motility is related to RhoA and Rac1 [77]. By knocking down Piezo1, GTP-Rac1 is activated, thus facilitating the formation of lamellipodia. It also interrupts the cycle of GTP-GDP transitions, suppresses Rho protein activation, and consequently causes a reduction in stress fibers in cells (e.g., F-actin). The loosening of F-actin was also obtained by transfection of gastric cancer cells with siPIEZO1. As a result of these actions (Piezo1 knockdown and siPIEZO1 transfection), the morphology of gastric cancer cells was different (the cells were elongated and acquired irregular shapes) and the cell motility was reduced, indicating that Piezo1 has an essential role in cancer migration [47]. Moreover, Piezo1 is blamed for the aberrant proliferative ability of gastric cancer cells, because the knocking down of Piezo1 causes significant G0/G1 arrest [77]. Furthermore, cell transfection with siPIEZO1 causes significantly decreased expression of the p53/p21 axis, Ki-67, CDK4, CDK6, and CyclinD1, which trigger proliferation. Piezo1 activation increases the mitochondrial membrane potential, which is responsible for many basic cell processes, including cell proliferation, but also gene transcription, differentiation, cell death, cancer metastasis, and angiogenesis [76]. Apoptosis of gastric cancer cells is also regulated by Piezo1. Flow cytometry assay results showed that the apoptotic activity of gastric cancer cells was inhibited after treatment with Yoda1 which activates Piezo1 [78]. Gastric cancer growth and its metastases are said to be controlled by Piezo1. Piezo1, through Ca^2+^ influx, upregulates the expression of hypoxia-inducible factor 1α (HIF-1α) which in turn induces the production of vascular endothelial growth factor (VEGF) and accelerates epithelial–mesenchymal transition (EMT). Consequently, angiogenesis, tumor growth, and, finally, invasion with metastasis are endorsed by Piezo1. The expression of Calpain 1/2 which is responsible for angiogenesis together with the division of focal adhesions, is induced by Piezo1 through activation of HIF-1α [76,77,78]. In the case of Piezo1 knockdown, cancer cells present an enhanced sensitivity to Cisplatin or 5-FU [79]. It is also worth mentioning that the Piezo1 chromosomal locus (16q24) when associated with the loss of heterozygosity contributes to the development of gastric cancer [83]. 

### 4.6. Bladder Cancer

Little is known about the involvement of Piezo1 and 2 in bladder cancer. However, Piezo 1/2 are expressed in bladder cells and even at higher levels in bladder cancer cells [47,84]. In a normal bladder, Piezo 1 is responsible for responding to mechanical stretch stimuli by causing Ca^2+^ influx and ATP release which lead to muscle contraction [85]. If this process plays a role in tumorigenesis remains unknown, yet the number of Piezo1/2 in bladder cancer cells is significantly high. So far, Piezo1 expression is correlated with tumor stage, grade, and size and is increased in ≥pT2. Piezo2 overexpression occurs only in ≥pT2 patients as compared to pT0, but no significant correlation with tumor grade was observed. In bladder cancer with lymph node metastasis as compared to specimens without lymph node metastasis, there are no differences in the expression of Piezo1/2 and no correlation with age and gender [84]. Although Piezo1/2 might play a role in bladder carcinogenesis, there is a need for more in-depth research to provide more evidence. 

### 4.7. Lung Cancer

The exception to the rule of Piezo1 supporting tumorigenesis is Piezo1’s role in lung cancer. In fact, there is more evidence of an omnidirectional role of Piezo1 in tumors. Piezo1 is highly expressed in lung tissue [86]. Nonetheless, it is depleted in lung cancer cells [87,88]. In cell lines of small cell lung cancer (SCLC), the reduced expression of Piezo1 was evidenced by RT-PCR [87]. However, it occurred that the low number of Piezo1 in SCLC does not reduce cell migration, although Piezo1 is known for being an activator of integrin–ligand affinity [89]. On the contrary, reduced expression of Piezo1 leads to a greater migration by a reduced integrin affinity to its ligand, cell adherence, and, consequently, anchorage-independent growth and amoeboid movement which is typical for SCLC metastasis [90,91,92]. As a confirmation of the amoeboid movement in Piezo1-depleted cancer cells, by downregulation of its marker, TNS4, the actin cytoskeleton becomes organized in ring-like structures, not in normal actin fibers, and the calpain activity involved in the integrin-dependent invasion/migration pathway is decreased. The loss of Piezo1 expression in the human bronchial epithelial cell line (16HBE) causes increased colony formation in soft agar [90]. Similar observations are reported for non-small cell lung cancer (NSCLC) which includes lung adenocarcinoma (LUAD) and lung squamous cell carcinoma (LUSC) [91]. Here, not only the expression of Piezo1 but also the one of Piezo2 is reduced. However, there is a difference between the mechanisms leading to Piezo downregulation; for Piezo1, it is a highly deep deletion rate of its gene and for Piezo2 it is a gene mutation [91]. Reduced Piezo1/2 expression in NSCLC also triggers cell migration. Moreover, NSCLC cells transfected with sh-Piezo1/2 (which knock down Piezo genes) show greater cell growth. These features influence the overall survival (OS) of patients with NSCLC. High expression of Piezo1/2 correlates with better OS, especially for patients with LUAD, but not for patients with LUSC. Piezo1/2 upregulation is also correlated with better OS in non-smoking patients, but not in patients who have smoked. Furthermore, Piezo1/2 has a more sensitive prognostic role in female patients than in male patients and in patients at earlier stages than in the latter ones [87,88]. These correlations clearly make Piezo1/2 expression a promising prognostic marker of OS in NSCLC in the future.

### 4.8. Leukemia

Functional Piezo1 channels are reported in leukemia cells (human myeloid leukemia K562 cell line), but their influence on tumor cell behavior remains unknown [62]. However, Piezo1 is surely activated there by its agonist, Yoda1, as patch-clamp measurements could verify in real time [93]. Other studies have shown that Piezo1 participates in maintaining volume homeostasis in erythrocytes and that Piezo1 gene mutation is frequently associated with dehydrated hereditary stomatocytosis [94,95]. Leukemia may be induced by in vitro erythroid differentiation, thus Piezo1 and its influence on cell volume could play an important role in the leukemic pathology occurrence which needs to be further investigated [96].

### 4.9. Esophageal Squamous Cell Carcinoma 

**Figure 3:**
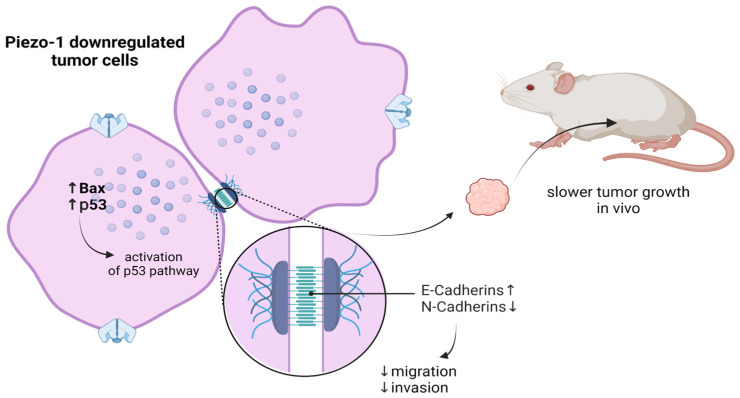
Piezo1 downregulation is linked to higher expression of p53 and Bax. Furthermore, there is the upregulation of E-cadherin and a decrease in N-cadherin expression. As a result, the inhibition of migration and the invasion of tumor cells occurs slowing down the growth of tumors in vivo [98].

Esophageal squamous cell carcinoma (ESCC) is the most common malignant neoplasm of this organ. During the swallowing process, the esophageal tissue is subjected to constant changes in mechanical forces. Interestingly, the expression of Piezo1 in esophageal cells is relatively higher than in the rest of the body’s tissues [97]. Gao L et al. [98] investigated the occurrence of these mechanosensitive channels in cancer cells and the effect of Piezo1 knockdown. The Piezo1 expression level was significantly increased in cancer compared to pre-cancerous tissues. Piezo1 downregulation reduces migration and invasion of cancer cells. This phenomenon may be explained by the upregulation of E-cadherin and a decrease in N-cadherin expression. A mutual connection between Piezo1 and the p53 signaling pathway has also been shown. Expression of p53 and Bax was clearly higher in the downregulated cells than in the control test. At the same time, it was proven that inhibition of p53 significantly upregulates Piezo1. Gao and collab. also assessed the behavior of ESCC cells in vivo. Piezo1-downregulated cells and control cells were injected into the subcutaneous tissues of mice. The results indicate that tumors in mice injected with Piezo1-silenced cells showed slower growth than those in control mice (Figure 3). 

### 4.10. Gliomas

The cellular component and the cerebrospinal fluid help to maintain the brain tissue mechanics in homeostasis. Mechanical forces are significantly reorganized under brain tumor conditions. How tumor cells sense and regulate tissue mechanics is largely unknown [40]. For this reason, attention is paid to mechanosensitive ion channels as potential regulators of the development of malignancies within the brain, especially gliomas, which are the most common primary central nervous system tumors [99,100,101,102]. Piezo1 is overexpressed in aggressive human gliomas and is associated with a poorer prognosis [101,102]. High expression levels are found in brain tumors with a high WHO grade (glioblastoma multiforme— GBM), IDH-wild-type status, and mesenchymal subtype [101]. It has been shown that the high tissue pressure generated by high-grade glioma provides mechanical stimuli for the Piezo1 channel. Subsequently, it plays an essential role in the proliferation and metastasis of cancer cells. Additionally, a correlation between Piezo1 upregulation and mutations in the TTN, PTEN, and NF1 genes was demonstrated [101]. Piezo1 overexpression has also been correlated with genes related to the tumor microenvironment, which are connected with extracellular matrix reorganization, cell adhesion, and angiogenesis [101]. Qu S et al. correlated Piezo1 overexpression and peritumoral brain edema (PTBE) in GBM patients. PTBE significantly escalates the patient’s symptoms and some studies suggest that it is an important prognostic factor. Piezo1 causes an intracellular cascade of events in endothelium cells that leads to the degradation of intercellular junctions (such as those based on E-cadherin, N-cadherin, and β-catenin) and the intensification of angioedema. PTBE can result in many potential risks, such as epilepsy, neurological dysfunctions, and an increase in intracranial pressure [100].

### 4.11. Prostate Cancer 

Prostate cancer (PCa) is one of the most common cancers in men worldwide [102]. It is a well-known fact that androgen receptors are expressed in both primary tumors and metastases [103]. Testosterone and its metabolites contribute to the growth and development of cancer [104]. However, its exact etiology is not completely understood. Studies have shown that the pressure in prostate tumors is notably higher than in non-cancerous tissues and may be responsible for resistance to apoptosis. A similar situation is observed in the metastasis of PCa to the bones. The pressure in the bone microenvironment promotes the growth of metastasis [105]. Han Y et al. [106] found that the expression of Piezo1 within tumors is significantly higher than in normal tissues. It was shown that downregulation of Piezo1 expression inhibits the proliferation and migration of cancer cells in vitro as well as the growth of tumors implanted into mice. The correlation between Piezo1 and the Act/mTOR pathway was also found in PCa. Han et al. considered that high expression of Piezo1 channels causes the calcium-dependent activation of Akt/mTOR. This results in increased expression of cyclin D1 and CDK4. Subsequently, activation of the cyclin D1/CDK4 complex may facilitate cell survival and cell cycle progression [106]. 

### 4.12. Colon Cancer 

Colon cancer (ColCa) is believed to arise from two types of precursor polyps via two independent pathways: conventional adenoma associated with a mutation in the APC gene and serrated adenoma with unknown genetic ethology [107]. Interestingly, Spier I. et al. suggested that a germinal mutation in the Piezo1 gene may be associated with a rare predisposition to form colon adenomas [108]. Hashimoto T et al. demonstrated the presence of the PIEZO1–RSPO2 fusion gene in a traditional serrated adenoma (TSA). However, the authors suggested that the activity of the PIEZO1 promoter, rather than its function, is important for this fusion [80]. Sun Y et al. reported that in ColCa, the Piezo1 channel is upregulated, and its high expression is associated with a poorer prognosis and vascular invasion. Moreover, Piezo1 overexpression promotes cell migration and metastasis. The study also showed a complex relationship of Piezo1 with proteins such as mitochondrial calcium uniporter (MCU), hypoxia-inducible factor 1 (HIF-1a), and vascular endothelial growth factor (VEGF). MCU and HIF-1a could be downstream targets of Piezo1. MCU is downregulated in ColCa cells. Furthermore, the knockdown of MCU is associated with the higher viability of cancer cells. HIF-1α is a calcium-sensitive factor that promotes tumor progression and is upregulated in ColCa. VEGF has been confirmed as a downstream target activated by HIF-1. The present study also demonstrated inhibition of HIF-1a and VEGF after Piezo1 silencing. In conclusion, San Y. et al. indicate that the Piezo1–MCU–HIF–1α–VEGF axis may play an important role in the development of colon cancer [109,110].

### 4.13. Osteosarcoma and Synovial Sarcoma

Mechanosensitive ion channel Piezo1 was discovered in mesenchymal tumors such as osteosarcoma (OsS) and synovial sarcoma (SynS) [111,112]. OsS is the most common type of bone tumor in children and adolescents [113,114]. Studies indicate that Piezo1 reduces apoptosis in human chondrocytes and OsS cells. Jiang L. et al. showed that Piezo silencing could inhibit the invasion of the OsS cells in vivo [111]. It has been shown that knocking down Piezo1 expression caused significantly lower cell viability in SynS cells [112]. Other mechanosensitive ion channels (TRPC4/C1) were also expressed in SynS. Muraki K et al. demonstrated that TRPC4/C1 activation causes a potent cytotoxic effect on SynS cells mediated via Na^+^ loading [115] (Table 1).

### 4.14. Angiogenesis

Both Piezo1 and Piezo2 are present in endothelial cells and play a role in the transduction of mechanical signals from the bloodstream to the cell triggering pleiotropic mechanisms. The activation of Piezo1 occurs through the stress forces exerted by blood flow on endothelial cells. The shear stress from non-laminar blood flow triggers Piezo1 via modulating membrane tension by a mechanism that is yet unknown [117]. Piezo1 then causes Ca^2+^ influx to the cell cytoplasm and, consequently, many endothelial repositionings, such as cell alignment and structural reorganization in the direction of flow, take place [118,119,120]. For the alignment, the proteolytic cleavage of the actin cytoskeleton and the focal adhesion proteins induced by shear-stress-enhanced Ca^2+^ entry are responsible, while for the cell structural reorganization, the calpain activated by Ca^2+^ is mainly responsible [118]. Piezo1 regulates the paradoxical migration of endothelial cells in static conditions through VEGF-evoked phosphorylation of eNOS at serine 1177 which enhances eNOS activity and therefore NO production [118]. Yet, shear stress activating Piezo1 causes the release of ATP from endothelial cells which induces the Gq/G11-coupled purinergic P2Y2 receptor and then eNOS together with AKT [121,122]. Although these mechanisms are complicated and still not fully known, Piezo1 plays an essential role in vascular architecture. Both Li J. et al. and Ranade S.S. et al. investigated mouse embryos in which Piezo1 was homozygotically deleted [118] or knocked down [119], with both actions resulting in a lethal effect. The cause of death was the abnormalities in embryos’ vascularization induced by the lack of Piezo1 and its stress activation. Vascular architecture is also regulated by Piezo1 in human umbilical vein endothelial cells (HUVECs) [118,120]. Here, Piezo1 induces migration of these cells towards vascular endothelial growth factor (VEGF), the main stimulant of angiogenesis in vivo [122,123]. 

A direct influence of Piezo on tumor angiogenesis is evidenced only in rat squamous cell carcinoma cells and glioma [76,124]. In the first case, Piezo1 was mechanically activated, then it triggered endothelin release from the stimulated cell, followed by activation of endothelin A receptors in the neighboring tumor cells. This suggests that endothelin signaling controlled by Piezo1 plays a role in cancer paracrine communication and metabolism [76]. In gliomas, however, it is Piezo2 that supports tumor angiogenesis. This effect is connected to Ca^2+^-dependent overexpression of Wnt11 which is released by endothelial cells and, finally, enhanced angiogenesis by β-catenin-dependent signaling occurs [124].

Taking into consideration how many pathways in angiogenesis are modulated by Piezo, it is important to broaden the research activities on this topic with an emphasis on tumor aspects.

### 4.15. Metastasis

The development of tumor metastasis is responsible for over 90% of cancer-related deaths and is associated with serious clinical issues [125]. Several hypotheses have been described to explain the origin of tumor metastases. These may include mesenchymal–epithelial transition, the accumulation of mutations in stem cells, the macrophage facilitation process, and macrophage origin, including transformation or fusion hybridization with neoplastic cells. Recently, there has been increasing interest in understanding how cancer cells convert mechanical into biochemical signals in tumor development and metastasis. Piezo1 activation and calcium as the Piezo1-dependent second messenger are described as one of the potential metastatic pathways [2]. In general, the metastasis process consists of five main steps: (1) invasion and migration, (2) intravasation, (3) dissemination, (4) extravasation, and (5) colonization [126].

### 4.16. Invasion and Migration 

Piezo1 activation promotes migration in breast, gastric, colorectal, pancreatic, and prostate cancer cells [127]. Before cancer cells begin to migrate, both cell conformational changes and changes in the tumor microenvironment must occur [91,128]. Piezo1 activation works on many levels, including regulation of gene transcription and increasing matrix stiffness. Piezo1 induces tissue stiffening through the binding of integrins and focal adhesions to stiffer matrixes, which is then associated with matrix remodeling and facilitates migration [126]. Epithelial-to-mesenchymal transition (EMT), a process by which epithelial cells undergo changes to become more mesenchymal in phenotype, is promoted by Piezo1. This process precedes migration [128]. Physiologically, EMT is an essential mechanism in embryonic development and tissue repair. On the other hand, EMT also contributes to the progression of disease, including cancer [129]. To effectively overcome the mechanical constraints imposed by the basement membrane and tissues, cancer cells can form structures called invadosomes that can both contact and break down the extracellular matrix through metalloprotease activity. Several key signaling pathways controlling invadosome functions are Ca^2+^-sensitive and Piezo1-dependent [126]. 

### 4.17. Intravasation and Extravasation 

Despite the fact that intravasation and extravasation take place at different stages of metastasis, their role is similar. Piezo1 plays a role in promoting angiogenesis, which allows the escape of cancer cells through leaky vasculature [127]. Matrix stiffness induced by Piezo1 promotes N-cadherin expression on endothelial cells, which supports a mesenchymal phenotype in cancer cells, allowing them to squeeze between cells during intravasation [127]. It has been shown that Piezo1 plays a role in the shear stress-induced release of ATP in red blood cells. Subsequently, paracrine signaling induces the formation of inter-endothelial junctions and supports the intravasation and extravasation of cancer cells [129]. 

### 4.18. Dissemination and Colonization

Various studies have shown that due to the influence of Piezo1 on the EMT process [130], this mechanosensitive channel could be considered one of the causes of cancer dissemination. In addition, it has been described that Piezo is a trefoil factor 1 (TFF1) binding protein [79]. Studies have shown that increased levels of TFF1 are associated with the promotion of dissemination in different cancers. Piezo1 has not been directly associated with colonization; however, increased intraosseous pressure has been shown to promote colonization of the bone by prostate cancer cells [127]. 

### 4.19. Myeloid-Derived Suppressor Cells 

**Figure 4:**
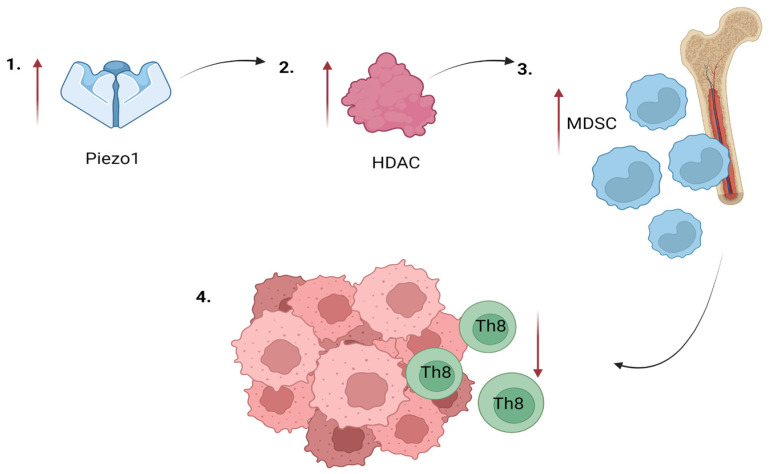
Piezo1 is involved in the activation of myeloid-derived suppressor cells (MDSCs): (**1**) Piezo1 overexpression, (**2**) HDAC hyperactivity, (**3**) MDSC recruitment, and (**4**) lower CD8+ T cell response in the tumor microenvironment [132,138].

Myeloid cells can sense mechanical forces via Piezo1 signaling. Piezo1-deficient leukocytes were unable to transduce mechanosensory signals. It has been shown that Piezo1 signaling in inflammatory cells promotes cancer progression and myeloid-derived suppressor cells (MDSCs) expansion [131]. MDSCs are immature myeloid cells with the ability to downregulate adaptive immune responses. The mechanisms of MDSC’s action are diverse and require either contact between cells or the release of soluble specific factors [132]. Previously, it has been shown that MDSCs are recruited into the microenvironment of pancreatic ductal adenocarcinoma by means of biochemical signals such as granulocyte–macrophage colony-stimulating factor (GM-CSF), chemokine signals consequent of Toll-like receptor 9 activation in pancreatic stellate cells, and soluble inflammatory mediators generated by the distinctive tumor-associated microbiome [133,134,135,136,137]. As a result, recruited MDSCs to decrease the action of CD8+ T cell responses in the tumor microenvironment (TME) (Figure 4). However, the mechanisms of the internal activation of MDSCs are not completely known [132]. Recent findings suggest that Piezo1 can activate histone deacetylases (HDACs), which are involved in regulating the function and differentiating of MDSCs. Studies have shown that inhibiting HDAC expression on MDSCs promoted their differentiation to less suppressive cells and reduced their immunosuppressive effect in the TME [138]. Aykut et al. demonstrated that HDAC2 is decreased in cells with downregulation of Piezo1 and mechanical stimulation promotes Piezo1-dependent myeloid cell expansion by suppressing the retinoblastoma gene Rb1. In conclusion, Piezo1 can be considered as a significant immunological checkpoint [131]. Authors should discuss the results and how they can be interpreted from the perspective of previous studies and of the working hypotheses. The findings and their implications should be discussed in the broadest context possible. Future research directions may also be highlighted.

## 5. TRP

### 5.1. Introduction to TRP

TRP is a type of mechanoreceptor widely expressed in particular tissues and displays numerous functions. Eight TRP families can be distinguished: TRPC (canonical), TRPV (vanilloid), TRPM (melastatin), TRPML (mucolipin), TRPP (polycystin), TRPA (ankyrin transmembrane protein), TRPN (NomPC-like), and the recently reported TRPY (yeast) [138]. Among them, TRPV, TRPM, and TRPC are the channels mostly correlated with malignant growth and progression [139].

### 5.2. TRPV Family

*TRPV1*

Sensory neurons and plasma membranes are the main tissue localization of TRPV1. This family of channels is sensitive to heat, pH, and compounds (e.g., capsaicin) and is also involved in thermal nociception [140,141,142,143]. In tumors, TRPV1 occurs in breast, prostate, and urothelial cancers, human papillary thyroid carcinoma, and also gliomas [84,142,144].

*TRPV2*

Oncogenicity in different types of cancers is associated with the expression of the TRPV2 channel [139]. It has been shown that TRPV2 expression is changed in hematological cancers, including mantle cell lymphoma, multiple myeloma, Burkitt lymphoma, acute myeloid leukemia, and myelodysplastic syndrome [139,145]. Overexpression of TRPV2 correlates with greater tumor progression and a poorer prognosis for glioblastoma multiforme patients [146]. Interestingly, higher recurrence-free survival is linked with greater expression of TRPV2 in triple-negative breast cancer (TNBC) and estrogen receptor β-(ERβ-)negative breast cancer patients who undergo doxorubicin chemotherapy [147]. Patients with esophageal squamous cell carcinoma harboring high expression levels of TRPV2 have a worse five-year overall survival rate after surgery when compared to patients with low TRPV2 expression [148]. The correlation between hepatocarcinogenesis, portal vein invasion, and TRPV2 expression is also observed [149]. Furthermore, TRPV2 oncogenicity in bladder cancer is shown, but it is not well understood. In this case, the oncogenic activity of TRPV2 is linked to the alteration of its expression profile [150].

*TRPV3*

TRPV3 is a non-selective Ca^2+^ channel that is highly expressed in epithelial tissues, especially in keratinocytes of the skin. There, TRPV3 can be triggered by heat and can regulate various phenomena such as skin barrier formation, wound healing, sensing of temperature, itching, and pain [141]. Little is known about TRPV3’s role in cancers. However, as it regulates the growth and survival of skin cells, its overexpression could be associated with the proliferation of lung cancer cells [151,152].

*TRPV4*

The high expression level of TRPV4 is especially characteristic for epithelial tissues, where TRPV4 reacts to, a.o. heat, osmotic changes, and mechanical stretching by inducing Ca^2+^ influx [147,153,154]. This influx has an essential role in cell volume regulation through the interaction between TRPV4 and F-actin [155,156] or the formation of intact cell–cell junctions in skin keratinocytes [157]. TRPV4 itself is responsible for vascular development, tone, and permeability of vessels [158,159]. In tumors, it influences angiogenesis and tumor vessel maturation by modulating tumor epithelial cells’ mechanosensitive ion channels (TEC). The lack or reduced expression level of TRPV4 is correlated with aberrant TEC to extracellular matrix stiffness, enhanced migration, and abnormal angiogenesis [160]. These phenomena are connected to the interaction of TRPV4 with either the Rho/Rho kinase pathway [161] or the ERK1/2 phosphorylation process [162]. Interestingly, in breast and renal-derived tumor endothelial cells, TRPV4 is upregulated [163]. After TRPV4 activation by arachidonic acid and a Ca^2+^ influx is produced, increased endothelial cell migration for breast tumor-derived endothelial cells is observed [164].

All the evidence suggests that the functionality of TRPV4 in tumor cells is truly complex and it needs to be further studied.

*TRPV5 and TRPV6*

The TRPV5 and TRPV6 families are very similar both in structure and their functions [165]. These channels are Ca^2+^-selective, responsive to 1,25-dihydroxyvitamin D3, and they regulate the homeostasis of Ca^2+^ in organisms. The role of TRPV5 in cancer is barely known, contrary to TRPV6 [166,167]. The expression of the latter is upregulated in prostate, breast, colon, esophageal, and cervical tumor tissues and also in the corresponding tumor cell lines. TRPV6 in tumors enhances metastasis and modulates chemotherapy resistance [167,168,169,170].

### 5.3. TRPM Family 

TRPM members are divided into four groups: TRPM1/M3, M2/M8, M4/M5, and M6/M7. TRPM channels are involved in plenty of physiological functions. However, they are also linked to diverse pathophysiological human processes, including cardiovascular and neurodegenerative alterations, organ dysfunction, and cancer [170]. When it comes to cancer, the most studied TRPM channel in relation to metastases is TRPM7. In breast cancer, diminished distant metastasis free-survival and free-recurrence survival are correlated to the upregulation of TRPM7 [171,172]. Moreover, the silencing of TRPM7 in the triple-negative breast cancer cells MDA-MB-231 decreased their metastatic potential in vivo, but not their viability [171]. Its overexpression is also evident in ductal adenocarcinoma cells and in areas of estrogen-receptor-negative invasive ductal cancer when a comparison to normal cells is made [173]. In pancreatic cancer, TRPM7 correlates with tumor size and stage and its overexpression is characteristic of metastasis [174,175]. Higher TRPM7 mRNA and protein expression is connected with bladder cancer and correlates with tumor recurrence, migration, invasion, metastasis, and a poorer prognosis [176]. TRPM7 is also overexpressed in nasopharyngeal tumors, and elevated TRPM7 expression is associated with worse prognosis and metastasis. It has been shown that TRPM7 knockdown in vitro decreases the migration and invasion of cancer cells and that TRPM7’s higher expression results in an increase in both migration and invasion in non-metastatic nasopharyngeal cancer cells [177]. Another important cancer TRPM family member is TRPM2. It is engaged in migration, invasion, tumor formation capacity, and epithelial–mesenchymal transition markers expression in gastric cancer cells [178].

### 5.4. TRPC Family

The seven mammalian TRPC members, which can be divided into four subgroups (TRPC1, TRPC2, TRPC4/5, and TRPC3/6/7) based on their structure and functional similarities, contribute to plenty of cellular functions and physiological roles [179]. TRPC5 is especially involved in cancer development. In colon cancer, TRPC5 expression is upregulated both in cancer tissue and in metastatic lymph nodes, which is associated with poorer overall survival and metastasis-free survival [180,181].

## 6. Conclusions

Mechanosensitive ion channels play a crucial role in the communication between cells and the surrounding environment. Therefore, they are essential for the adaptation and survival of cells—the cancer ones as well. The most important mechanosensitive ion channels for tumors are the Piezo and TRP families. TRPV, TRPM, and TRPC are the families mostly associated with tumor development. They play a wide role in the development of many types of cancers such as breast, prostate, and colon cancer. They affect tumor growth and spread on multiple levels.

Piezo 1 and 2 channels, although nowadays widely studied, influence cells by diverse mechanisms which are yet to be known in depth. In breast cancer cells, there is a high level of Piezo expression. The channels’ activation enhances tumor invasion and confined migration while it suppresses unconfined migration. The influence on proliferation is inconclusive. In oral cancer, Piezo induces tumor proliferation via Hippo and Yap signaling. In gastric cancer, there are numerous pathways connected to Piezo1 and its involvement in tumor proliferation, migration, invasion, and angiogenesis. The most essential is said to be Piezo 1’s correlations with integrin β1, RhoA, Rac1, and HIF-1α. The exact mechanisms of Piezo1 on bladder cancer cells are yet to be discovered; however, this channel has a positive impact on tumor stage, grade, and size. Piezo’s incoherent role in cancer is presented in lung cancer. In this cancer, it is Piezo inhibition, not activation, that supports tumor migration. In the case of esophageal squamous cell carcinoma, the downregulation of Piezo1 reduces the migration and invasion of cancer cells. Moreover, the expression of Piezo1 is associated with the activity of the TP53 pathway. The inhibition of TP53 significantly upregulates Piezo1. The expression of Piezo1 within prostate tumors is significantly higher than in normal tissues. In this cancer, the mechanism is closely associated with higher tissue pressure in the tumor and in bone metastasis. A germinal mutation in Piezo1 can cause the formation of polyps in the large intestine. Furthermore, the expression of Piezo1 in colon cancer is significantly higher and associated with a poorer prognosis. Piezo1 is overexpressed in aggressive human gliomas and it is associated with a poorer prognosis. Moreover, a correlation between Piezo1 upregulation and mutations in genes related to extracellular matrix reorganization, cell adhesion, and angiogenesis was also shown. The association between aggressive sarcomas such as synovial and osteosarcoma and Piezo1 expression was also demonstrated.

The family of Piezo mechanosensitive channels is essential in the development of normal as well as abnormal vessels. The role of Piezo in tumor angiogenesis has been so far evidenced in rat squamous cell carcinoma, where endothelin occurs, and gliomas, where β-catenin-dependent signaling is essential. It has also been shown that Piezo1 plays a crucial role in the development of cancer metastases on multiple levels of this process. Furthermore, Piezo1 can be a very important immunological checkpoint. Leukocytes without Piezo1 expression were unable to transduce mechanosensory signals. Moreover, it has been demonstrated that Piezo1 signaling in inflammatory cells promotes cancer progression and myeloid-derived suppressor cell expansion. 

The connection between mechanosensitive ion channels and cancer development is wide, multi-faceted, complex, and not fully explored. Further studies in this area are necessary to find among them new, interesting, and important therapeutic targets for modern oncology. 

## Figures and Tables

**Table 1:** The influence of Piezo activation on different cancer cells.

Cancer	Protumorigenic Effect	Antitumorigenic Effect	Additional Information
Breast cancer	Invasion, confined migration, proliferation [1,64,66]	Unconfined migration, proliferation [64,66]	
Oral cancer	Proliferation [67,68,69,70,71,72,73,74,75]		
Laryngeal cancer	Migration [76]		
Gastric cancer	Invasion, migration, proliferation, angiogenesis [77,78,79,80,81,82]		
Bladder cancer	Invasion, proliferation, migration [48,84,85]		
Lung cancer		Higher overall survival rate [88]	Piezo inhibition supports tumor migration [87,88,89,90,91,92,93,94]
Leukemia	Maintaining volume homeostasis in erythrocytes [93,94,95,96]		
Glioma	Proliferation, metastasis, angiogenesis, extracellular matrix reorganization [100,101]		The correlation between Piezo1 overexpression and peritumoral brain edema [100]
Esophageal Squamous Cell Carcinoma	Invasion, migration [98]		Piezo1 expression is linked with the TP53 pathway [98]
Prostate cancer	Proliferation, migration, tumor growth [105]		Piezo1 overexpression causes activation of the Akt/mTOR pathway [105]
Colon cancer	Migration, metastasis, vascular invasion [108,116]		The connection between Piezo1 and MCU, HIF-1a and VEGF [109,110]
Osteosarcoma	Apoptosis reduction, invasion [111]		
Synovial sarcoma	Increasing cancer cell viability [112]		

## Data Availability

Not applicable.
